# Genetic Base of Behavioral Disorders in Mucopolysaccharidoses: Transcriptomic Studies

**DOI:** 10.3390/ijms21031156

**Published:** 2020-02-10

**Authors:** Karolina Pierzynowska, Lidia Gaffke, Magdalena Podlacha, Grzegorz Węgrzyn

**Affiliations:** Department of Molecular Biology, University of Gdansk, Wita Stwosza 59, 80-308 Gdansk, Poland; karolina.pierzynowska@biol.ug.edu.pl (K.P.); lidia.gaffke@phdstud.ug.edu.pl (L.G.); magdalena.podlacha@ug.edu.pl (M.P.)

**Keywords:** mucopolysaccharidosis, transcriptomics, behavioral disorders

## Abstract

Mucopolysaccharidoses (MPS) are a group of inherited metabolic diseases caused by mutations leading to defective degradation of glycosaminoglycans (GAGs) and their accumulation in cells. Among 11 known types and subtypes of MPS, neuronopathy occurs in seven (MPS I, II, IIIA, IIIB, IIIC, IIID, VII). Brain dysfunctions, occurring in these seven types/subtypes include various behavioral disorders. Intriguingly, behavioral symptoms are significantly different between patients suffering from various MPS types. Molecular base of such differences remains unknown. Here, we asked if expression of genes considered as connected to behavior (based on Gene Ontology, GO terms) is changed in MPS. Using cell lines of all MPS types, we have performed transcriptomic (RNA-seq) studies and assessed expression of genes involved in behavior. We found significant differences between MPS types in this regard, with the most severe changes in MPS IIIA (the type considered as the behaviorally most severely affected), while the lowest changes in MPS IVA and MPS VI (types in which little or no behavioral disorders are known). Intriguingly, relatively severe changes were found also in MPS IVB (in which, despite no behavioral disorder noted, the same gene is mutated as in GM1 gangliosidosis, a severe neurodegenerative disease) and MPS IX (in which only a few patients were described to date, thus, behavioral problems are not well recognized). More detailed analyses of expression of certain genes allowed us to propose an association of specific changes in the levels of transcripts in specific MPS types to certain behavioral disorders observed in patients. Therefore, this work provides a principle for further studies on the molecular mechanism of behavioral changes occurring in MPS patients.

## 1. Introduction

The group of lysosomal storage diseases (LSD) [1] in which glycosaminoglycans (GAGs) are accumulated due to mutations in genes coding for enzymes involved in degradation of these complex carbohydrates is called mucopolysaccharidosis (MPS) [2]. There are 11 types and subtypes of MPS that are classified according to the kinds of stored GAGs and dysfunctions of specific enzymes [2]. In MPS types I and II, heparan sulfate and dermatan sulfate accumulate, but their degradation is inhibited at various stages due to dysfunctions of different enzymes [1]. Heparan sulfate is the primary GAG stored in MPS III, however, there are four subtypes of this disease (named A, B, C and D) which correspond to various defective enzymes responsible for degradation of this GAG; in addition, less efficient secondary storage of dermatan sulfate has also been reported in MPS III [3]. In MPS IV, keratan sulfate and chondroitin sulfate cannot be efficiently degraded due to defects in two different enzymes which is the basis to distinguish two subtypes of this MPS, called A and B. The sole GAG stored in MPS VI is dermatan sulfate, while heparan sulfate, dermatan sulfate and chondroitin sulfate accumulate in MPS VII. Finally, hyaluronic acid is stored in MPS IX (Table 1).

In the case of severe phenotypes, MPS patients can be diagnosed at the age of several months. However, usually, the diagnosis is made at the age of several years, sometimes after a long searching for identification of actual disease due to many unspecific symptoms occurring in patients [2]. Each MPS type has a progressive course, though the time of appearance of particular symptoms, their severity and progression rates differ considerably not only between MPS types but also within each type as variability among patients is huge [2].

MPS are severe diseases with serious dysfunctions of vast majority of tissues and organs [2]. However, behavioral symptoms are characteristic for only some MPS types in which various neuronopathic features occur [4]. Analysis of the storage material and occurrence of behavioral disorders indicates that such problems are characteristic for MPS types in which heparan sulfate is accumulated (Table 1). Therefore, it is evident that this GAG is responsible for neurodegeneration and behavioral disturbance in MPS. On the other hand, as indicated in Table 1, specific behavioral disorders are characteristic for certain MPS types. Although differences in behaviors of patients suffering from various MPS types are obvious, mechanisms leading to such specificities remain unknown. For example, although the same kinds of GAGs are stored in MPS I and II, patients suffering from the former type are generally gentle, calm, placid, and over-careful while those with the latter type develop an aggressive-like behavior. MPS III patients are hyperactive, aggressive and they reveal reduced fear [6,7,8,9,10,11].

Some attempts to understand molecular basis of differences between behaviors of patients with different MPS types have been published [12]. It was proposed that specific chemical moieties which are present at the ends of incompletely degraded heparan sulfate molecules can be responsible for different behaviors due to reactions with other compounds, crucial for signals determining neuronal functions [12]. Despite this hypothesis, specific mechanisms of the behavioral specificities of different MPS types are completely unclear. Therefore, in this work, using the RNA-seq technique, we have analyzed expression of genes potentially involved in behavior (based on QuickGO database, as defined by the Gene Ontology Consortium). We have used cell lines derived from patients with all MPS types to learn about differences in levels of transcripts of these genes which can be the first step in understanding molecular mechanism of behavioral disorders in these diseases. 

## 2. Results

In this study, we investigated cultured fibroblast lines derived from patients suffering from all known types and subtypes of MPS (i.e., MPS I, II, IIIA, IIIB, IIIC, IIID, IVA, IVB, VI, VII, IX). Wild-type fibroblast line, HDFa, has been used as a control. In addition, in some RT-qPCR experiments, two other fibroblast lines were used as controls (Table 2).

The cells were cultured as described in Section 4. Following isolation of total mRNA, transcriptomic analysis has been performed, employing the RNA-seq technology, as described in Section 4. For each MPS line and the control cell line, four biological repeats were used (each cell line was used to establish four independent cell cultures, each at different passage; the fibroblasts were between 4th and 15th passages), and in each experiment, number of reads was between 40 and 62 million.

**Figure 1:**
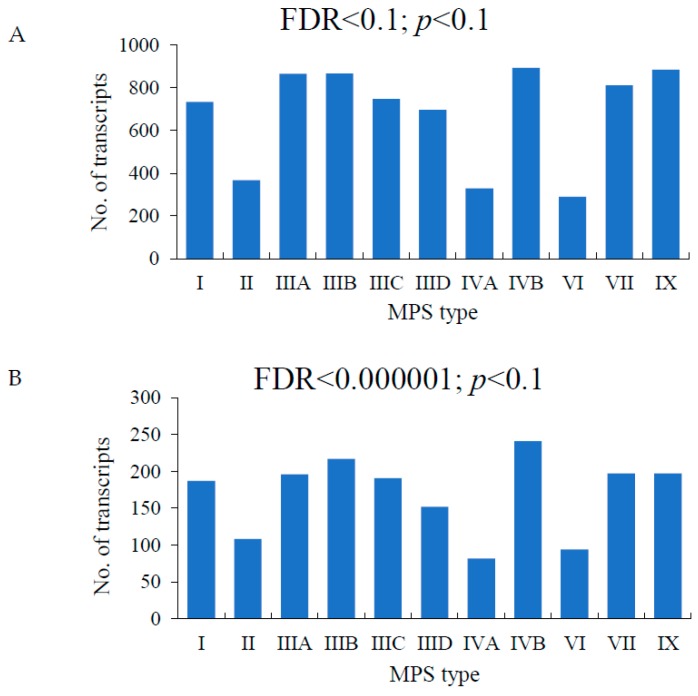
Number of transcripts with changed levels of expression (at FDR < 0.1; *p* < 0.1 (**A**) or FDR < 0.000001; *p* < 0.1 (**B**)) in different types of MPS relative to control cells (HDFa). FRD—false discovery rate; MPS—mucopolysaccharidosis.

When considering total transcriptome, number of transcripts in each MPS type with levels significantly changed relative to the control, at false discovery rate (FDR) < 0.1 and *p* < 0.1 (standard parameters for preliminary transcriptomic analyses with at least four biological repeats), was from almost 300 to almost 900 (Figure 1), indicating global changes in gene expression pattern in every disease. When more strict analysis conditions were employed, i.e. FDR < 10^−6^, these numbers were between 82 and 241, depending on MPS type (Figure 1) which still indicated a relatively high level of transcriptomic changes. 

Nevertheless, when in the next step we have focused on genes involved in behavior, selected according to the QuickGO database (term GO:0007610—behavior), following recommendations of the Gene Ontology Consortium, FDR < 0.1 and *p* < 0.1 were chosen to assess possibly global picture of transcriptomic changes. In this analysis, we have identified different numbers of such behavior-related genes which expression is changed in specific MPS type vs. control cell line (Table 3). Both up-regulated and down-regulated genes were found. However, differences between MPS types were considerable. The highest number of behavior-related genes with changed expression (relative to HDFa control) was found in MPS IIIA (39 genes), and the lowest number was detected in MPS VI (8 genes). It is worth to note that Sanfilippo disease (MPS III, including the MPS IIIA subtype) is the MPS type in which the most severe behavioral disturbances are described [13,14,15], while no behavioral abnormalities occur in MPS VI [16]. Therefore, we assume that, although our transcriptomic experiments were performed with RNA isolated from fibroblasts, not neurons, information about the expression of genes involved in behavioral processes gained from such studies can be accurate. Such distribution of changes in levels of transcripts coding for proteins taking part in various behavioral processes suggests that these results may be considered reliable in general analysis of molecular mechanisms of behavioral disturbances. As could be expected, levels of relatively high number of the studied transcripts were changed in other MPS III subtypes, MPS I and MPS VII (more than 20 in each case) which are known from behavioral disturbance. On the other hand, the MPS II type, in which behavioral changes are severe, was characterized by only 11 transcripts changed relative to control cells. Surprisingly high numbers of changes were found in MPS IVB and MPS IX. The MPS IVB subtype is described as a non-neurological disease, however, it is worth noting that dysfunction in the same enzyme, β-galactosidase, is responsible for both MPS IVB and GM1 gangliosidosis, a severe neurodegenerative disease, while positions of mutations in the *GLB1* gene determine the disease symptoms [17]. Regarding MPS IX, only a few patients have been described to date [2,18], thus, any conclusions on behavioral disorders in this disease are not substantiated. Therefore, analysis of changes in expression of genes related to behavior may be particularly interesting in this MPS type, providing some preliminary suggestions for further observations of patients.

**Figure 2:**
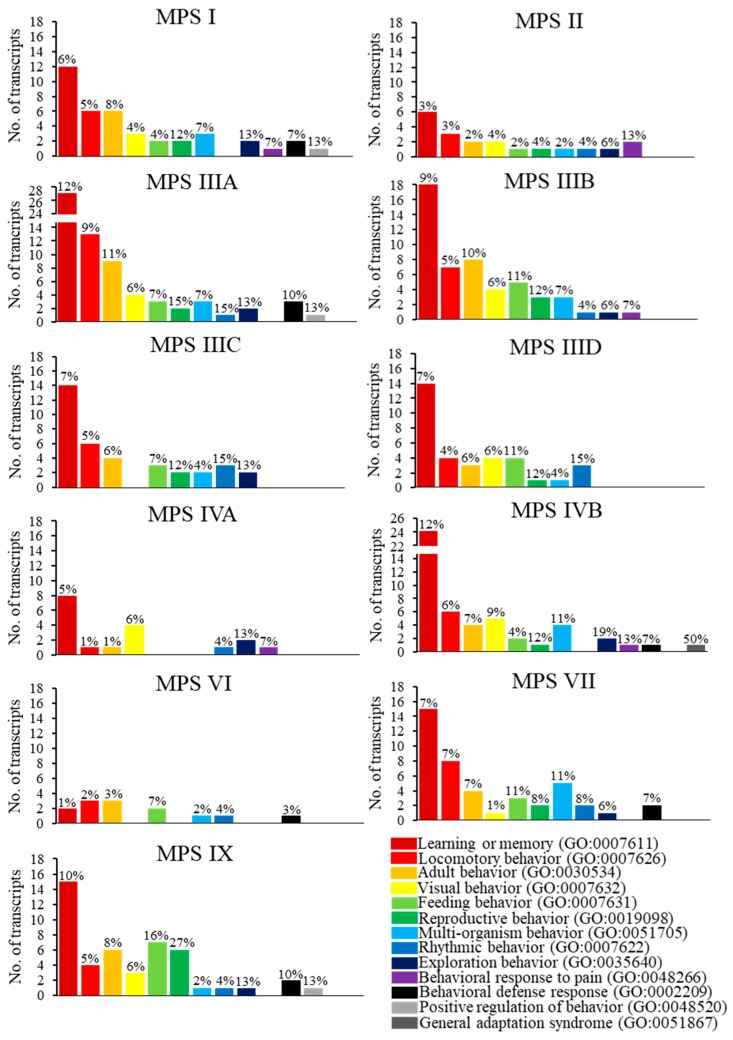
Number of transcripts, corresponding to genes from indicated sub-processes (child processes) of behavior (term GO:0007610), defined according to the QuickGO database, which levels were significantly changed in MPS cells (MPS type or subtype is indicated in each panel) relative to the control cells. Fractions (%) of genes represented in each given GO term being differentially expressed relative to HDFa are also demonstrated above corresponding columns. Each sub-process is indicated by different color, according to the legend presented in the right-lower corner.

We have asked what specific behavioral processes are changed at the gene expression level in all MPS types. Therefore, direct children terms of GO:0007610 (behavior) were analyzed. Significant changes in the expression of behavior-related genes were observed in following sub-processes: learning or memory (GO:0007611), locomotory behavior (GO:0007626), adult behavior (GO:0030534), visual behavior (GO:0007632), feeding behavior (GO:0007631), reproductive behavior (GO:0019098), multi-organism behavior (GO:0051705), rhythmic behavior (GO:0007622), exploration behavior (GO:0035640), behavioral response to pain (GO:0048266), behavioral defense response (GO:0002209), positive regulation of behavior (GO:0048520), and general adaptation syndrome (GO:0051867) (Figure 1). Interestingly, the highest number of changed transcripts in MPS I, MPS II, MPS III (all subtypes), MPS IV (both subtypes), MPS VII and MPS IX belonged to the sub-process of learning and memory (Figure 2). This correlates well with symptoms occurring in neuronopathic MPS types in which learning problems and memory deficits belong to the most characteristic features. The presence of changed expression of such genes in MPS IVA and MPS IVB is surprising, as no learning- or memory-related problems are considered as specific for these diseases. On the other hand, in another non-neuronopathic type, MPS VI, expression of only a few genes related to behavior was different than in the control cells (Figure 2) which correlates well with a lack of behavioral problems in this disease. This is also the case in MPS IVA, where significant number of considerably changed transcripts of behavior-related genes could be found only in the learning and memory sub-process but not in other sub-processes (Figure 2). In neuronopathic MPS types (MPS I, MPS II, all subtypes of MPS III, and MPS VII), the second group of genes for which number of transcripts with significantly changed levels was especially high was that described as locomotory behavior in the QuickGO database (Figure 2). This also correlates well with symptoms observed in patients, as various behavioral problems related to locomotion were described for MPS patients, including hyperactivity, pointless movement and not paying attention on obstacles on the way. There were no other clear preferences in groups of behavior-related genes with significant changes in expression between MPS cell lines and control fibroblasts, as in the rest of the subprocesses, with levels of only a few kinds of transcripts considerably changed (Figure 2). 

**Figure 3:**
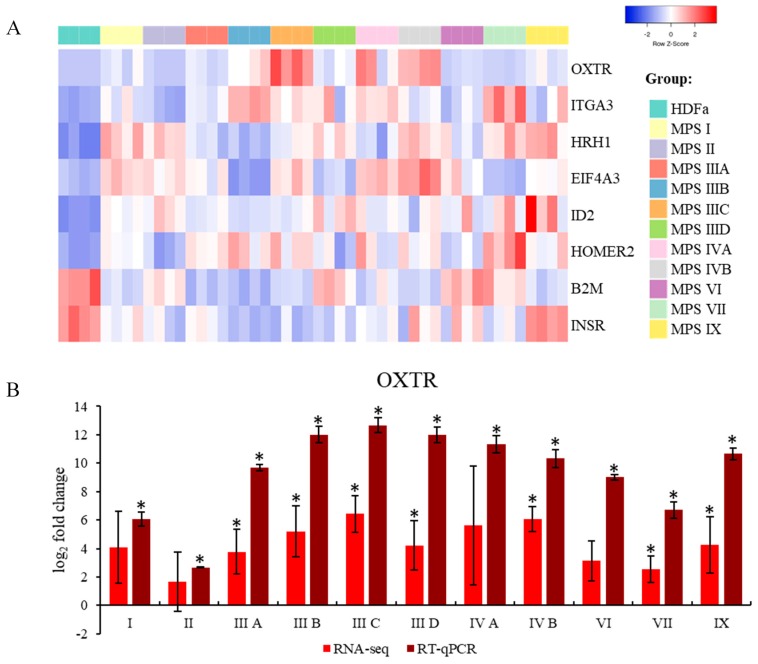
Genes which expression is significantly changed in at least six MPS types/subtypes relative to the control cells. Heat map of transcript levels is presented in panel (**A**) (MPS types and biological repeats are indicated). Comparison of results obtained for the *OXTR* gene using RNA-seq and RT-qPCR methods is shown in panel (**B**). Presented values are mean values from 4 experiments with SD shown as error bars. Statistically significant differences relative to control (HDFa) cells are indicated by asterisks.

We have asked what behavior-related genes were characterized by expression changed significantly in the most of MPS types. We found that expression of 8 such genes was considerably changed in at least 6 MPS types (Figure 3A and Table 4). The following genes were up-regulated: *OXTR* (coding for oxytocin receptor)*, ITGA3* (coding for integrin subunit alpha 3)*, HRH1* (coding for histamine receptor H1)*, EIF4A3* (coding for eukaryotic translation initiation factor 4A3)*, ID2* (coding for inhibitor of DNA binding 2)*,* and *HOMER2* (coding for Homer scaffold protein 2), while expression of *B2M* (coding for β-2-microglobulin) and *INSR* (coding for insulin receptor) was down-regulated in most of MPS types. Importantly, in MPS VI, it was only one gene (*ID2*) belonging to this group, corroborating previous indications that behavior-related genes are generally normally expressed in this non-neuronopathic disease. To test if the RNA-seq results are reliable, we have chosen the *OXTR* gene and investigated levels of its mRNA in all MPS types using RT-qPCR. Results of RNA-seq and RT-qPCR were compatible in all cases, with the same tendency in all tested cell lines, despite more pronounced differences observed in each MPS type vs. control cells (Figure 3B). These results indicate that values obtained in the RNA-seq analysis are reliable and can be interpreted with a high level of consistency.

**Figure 4:**
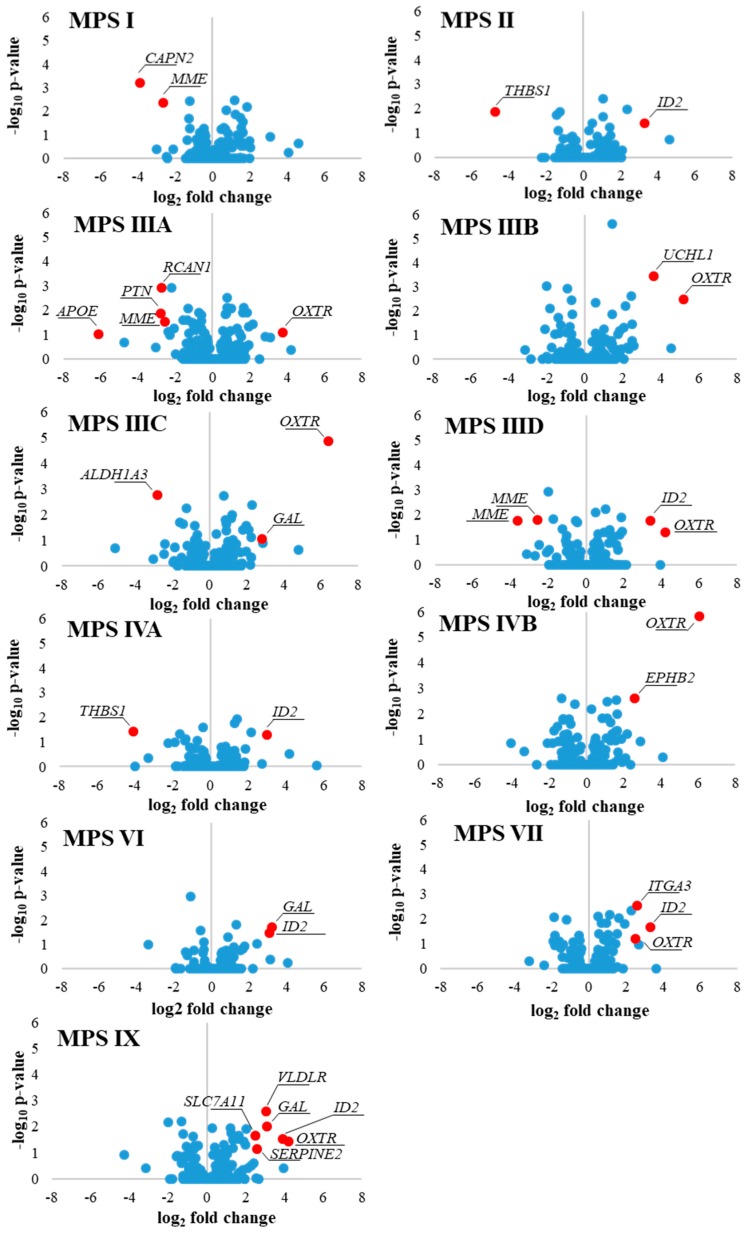
Volcano plots indicating genes which expression is significantly changed in MPS types relative to control cells (HDFa) and reached log_2_FC > 2.5. The gene (*MME*) for which two alternative transcripts were changed in the above manner is indicated in MPS IIID.

**Figure 5:**
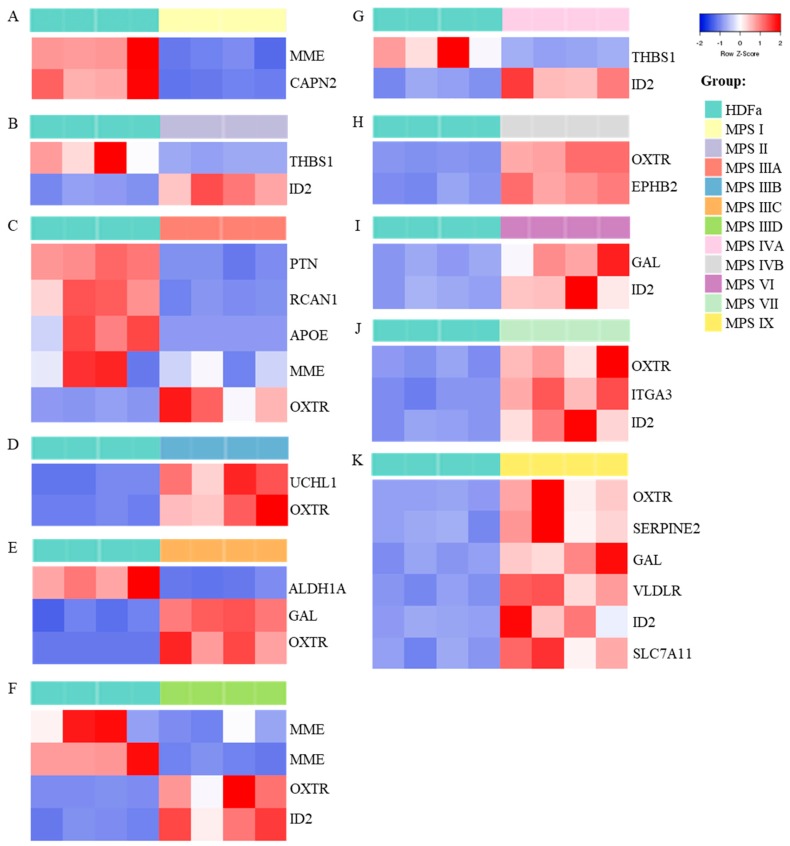
Genes which expression is particularly significantly changed (log_2_FC > 2.5) in different MPS types/subtypes relative to control cells – heat maps. Heat maps of transcript levels are presented for each MPS type (types: I, II, IIIA, IIIB, IIIC, IIID, IVA, IVB, VI, VII and IX in panels **A**–**K**, respectively), relative to control cell line (HDFa), with 4 biological repeats indicated in each case. Names of genes are indicated at the right side of each panel. The scale of changes in shown below panel K.

**Figure 6:**
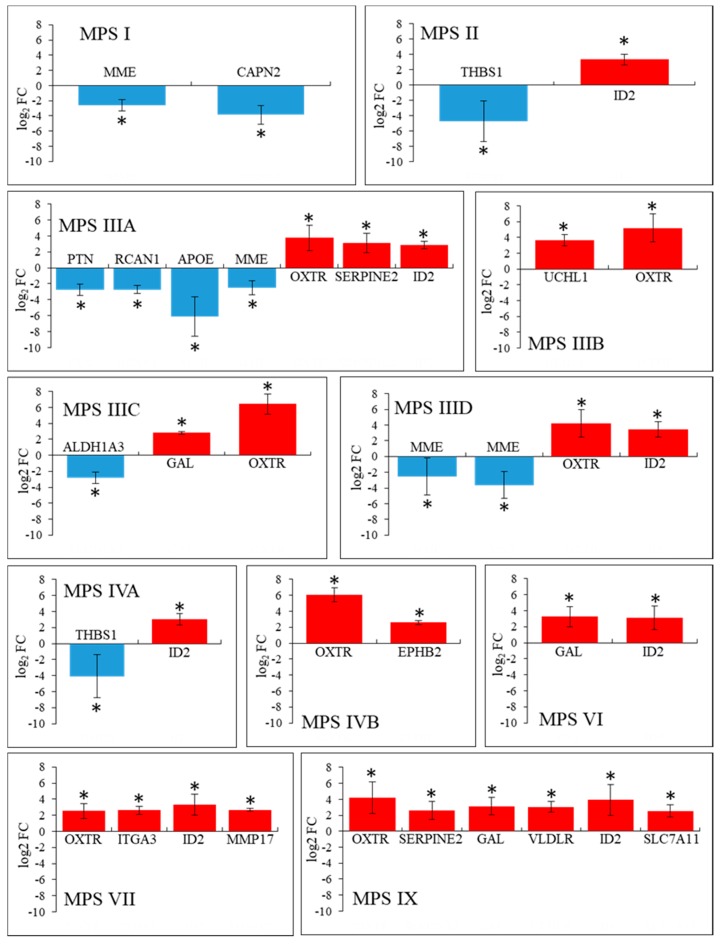
Genes which expression is particularly significantly changed (log_2_ FC > 2.5) in different MPS types/subtypes relative to control cells—quantitative analysis. Changes in levels of particular transcripts are presented for each MPS type relative to control cell line (HDFa). Names of genes are indicated. Down-regulation is marked in blue, and up-regulation in red. Presented values are mean values from 4 experiments with SD shown as error bars. Statistically significant differences relative to control (HDFa) cells are indicated by asterisks.

The results presented in Figure 3 indicted that there are some common features in patterns of expression of behavior-related genes in MPS, namely, expression of eight such genes (*OXTR, ITGA3, HRH1, EIF4A3, ID2, HOMER2*, *B2M,* and *INSR*) was significantly changed in six or more MPS types/subtypes. In the next step, we asked what genes are characterized by particularly significant changes in expression in each MPS type relative to control cells. In this analysis, the threshold was assessed to be log_2_ fold change (FC) > 2.5. The results are presented as volcano plots (Figure 4), heat maps (Figure 5), and column pictures (Figure 6). In volcano plots (Figure 4), the genes selected on the basis of FC versus *p* value analysis are indicated. Following genes reached the criterium of FC > 2.5 and statistically significant difference relative to control in particular MPS types: in MPS I, *MME* (coding for membrane metalloendopeptidase)*, CAPN2* (coding for calpain 2) (down-regulated) and *ID2* (up-regulated); in MPS II, *THBS1* (coding from thrombospondin 1) (down-regulated) and *ID2* (up-regulated); in MPS IIIA, *PTN* (coding for pleiotrophin)*, RCAN* (coding for regulator of calcineurin 1)*, APOE* (coding for apolipoprotein E)*, MME* (down-regulated) and *OXTR* (up-regulated); in MPS IIIB, *UCHL1* (coding for ubiquitin *C*-terminal hydrolase L1)*, OXTR* (up-regulated); in MPS IIIC, *ALDH1A3* (coding for aldehyde dehydrogenase 1 family member A3) and *GAL* (coding for galanin and GMAP prepropeptide)*, OXTR* (up-regulated); in MPS IIID, *MME* (down-regulated) and *OXTR, IDS2* (up-regulated); in MPS IVA, *THBS1* (down-regulated) and *ID2* (up-regulated); in MPS IVB, *OXTR, EPHB2* (coding for ephrin receptor B2) (up-regulated); in MPS VI, *GAL, ID2* (up-regulated); in MPS VII, *OXTR, ITGA3, ID2* (up-regulated); in MPS IX, *OXTR, SERPINE2* (coding for serpin family E member 2)*, GAL, VLDLR* (coding for very low density lipoprotein receptor)*, ID2, SLC7A11* (coding for solute carrier family 7 member 11) (up-regulated). The performed analyses allowed us also to consider alternative transcripts for particular genes. We found that the only gene with alternative transcripts which reached log_2_ FC > 2.5 and statistically significant difference relative to control was *MME* in MPS IIID (indicated in Figure 4). The potential meaning of changes in the expression of each gene is analyzed and discussed in Section 3.

**Figure 7:**
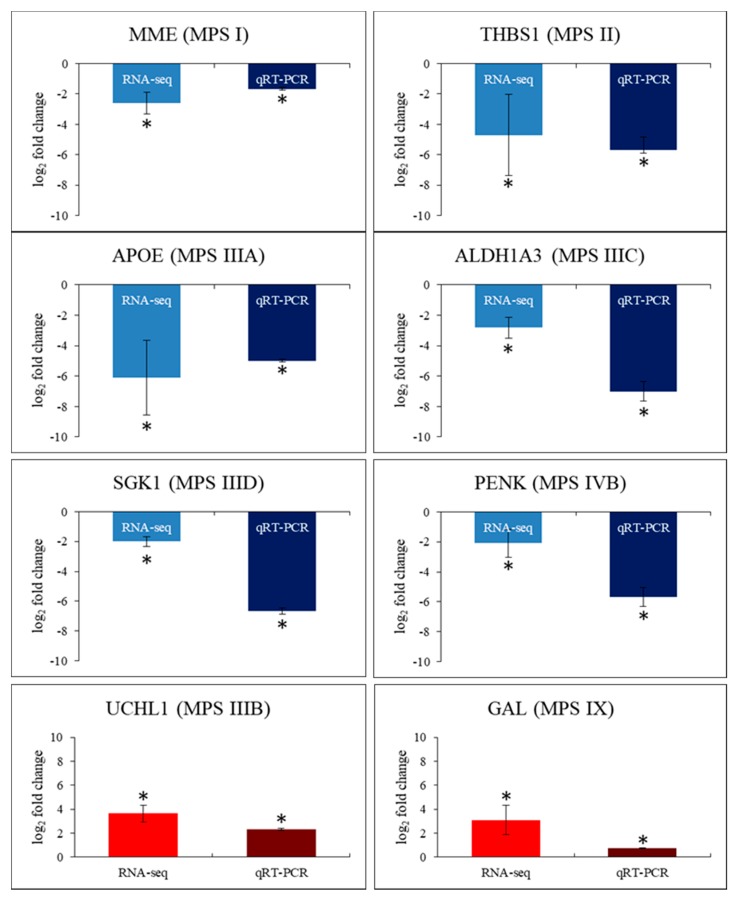
Comparison of results of assessing levels of transcripts using RNA-seq and RT-qPCR methods, obtained for selected genes in all tested MPS types/subtypes relative to control cell line (HDFa). One gene was tested in each MPS type/subtype as indicated in the figure. Presented values are mean values from 4 experiments with SD shown as error bars. Statistically significant differences relative to control (HDFa) cells are indicated by asterisks.

To test if RNA-seq results are reliable, we have analyzed mRNA levels of one gene in each MPS type/subtype using RT-qPCR. Results presented in Figure 7 demonstrate that RNA-seq and RT-qPCR data are compatible, as trends in expression changes were the same for all tested genes in all MPS types/subtypes. This indicates that analyses performed on the basis of RNA-seq data can be considered reliable. 

**Figure 8:**
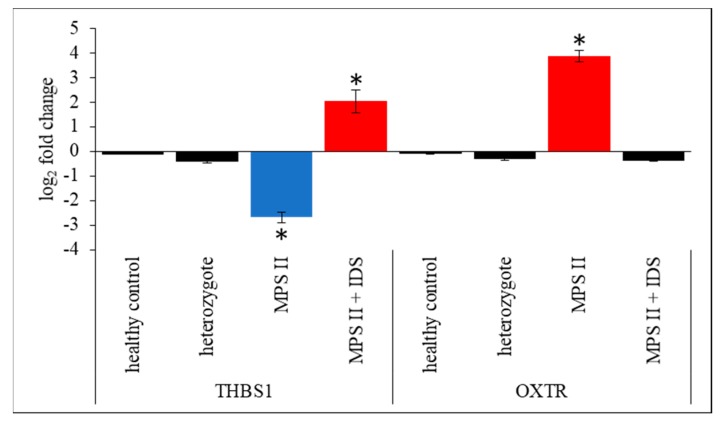
RT-qPCR analysis of levels of transcripts of selected genes (*THBS1* and *OXTR*) in control 2 (healthy female) and control 3 (*IDUA* heterozygote) fibroblasts, as well as in MPS II fibroblast, either untreated or treated with human recombinant iduronate-2-sulfatase (Elaprase, IDS) at 0.5 mg/mL for 24 h, relative to HDFa cells. Presented values are mean values from 3 experiments with SD shown as error bars. Statistically significant differences relative to control cells (HDFa) are indicated by asterisks.

In addition, we have used two more control fibroblast lines, one derived from a healthy female (control line 2; to assess any potential sex-related differences in gene expression), and another derived from *IDUA* heterozygote (control line 3; bearing a mutation p.Δ349 in one allele). When testing both additional controls, levels of transcripts of genes randomly chosen from those which revealed significant differences in MPS fibroblasts, *THBS1* and *OXTR*, were not significantly changed relative to HDFa cell line (Figure 8). These results corroborated the conclusion that changes in gene expression levels observed in MPS cells are specific for the disease rather than arising from random fluctuations among persons in general population (including sex- and heterozygote-related fluctuations). Moreover, when MPS II fibroblasts were treated with an enzyme used in enzyme replacement therapy for this MPS type, human recombinant iduronate-2-sulfatase (Elaprase), to normalize GAG levels in cells, the effects of changes in levels of *THBS1* and *OXTR* transcripts were reversed (Figure 8) which again supported the above conclusion.

## 3. Discussion

Behavioral changes are common in neuronopathic forms of MPS (MPS I, MPS II, MPS III, and MPS VII), however, their molecular mechanisms remain unclear [1,2,3,4,5,6,7,8,9,10,11]. It is generally accepted that the storage of heparan sulfate (HS) is responsible for these changes, as this GAG accumulates, between others, in neurons, contrary to other major GAGs stored in MPS cells [1,2,12]. On the other hand, among MPS types in which HS storage occurs, behavioral changes are very different [2,3,4,5,6,7,8,9,10]. Although some hypotheses were proposed on biochemical bases of these differences [12], it is still unknown how specific can behavioral changes be caused by the presence of accumulated molecules in lysosomes. 

Previous studies suggested that significant changes may occur in transcriptomes of MPS model animals [19]. Therefore, in this work, we have asked if there are specific changes in expression of genes associated with behavior. We have tested cell lines derived from patients suffering from all MPS types and subtypes, to obtain the whole picture of transcriptomic changes in this disease. Human cell lines, rather than animal models, have been used in order to assess changes correlated with human behavior which has specific features, often not represented in animals. 

Although we have used patient-derived fibroblast lines, not neurons, general analyses of transcriptomes, when considering genes included in the QuickGO database under the term behavior (GO:0007610), according to the Gene Ontology Consortium, indicated that the highest number of transcripts with changed levels relative to the control cell line (HDFa) occurred in MPS IIIA and the lowest number in MPS VI (Table 3). Since MPS III (Sanfilippo disease) is the MPS type in which the most severe behavioral changes occur, and no disease-specific behavioral abnormalities were described MPS VI, these results suggested that such analysis may reflect, at least to some extent, changes in regulations of gene expression which might be responsible for or connected to problems with various behavioral symptoms characteristic for MPS. More detailed analyses, including assessment of genes which expression is changed in particularly significant manner (Figure 2, Figure 3, Figure 4, Figure 5 and Figure 6), confirmed this general trend, and encouraged us to perform more detailed analyses.

While the simultaneous (i.e., in one study) use of the same kind of cells (fibroblasts) derived from patients suffering from all types and subtypes of MPS is an advantage of this study, there are obvious limitations which must be considered. Apart of the above mentioned simplification of the model, i.e., the use of fibroblasts instead of neural cells (which, on the other hand, allowed us to investigate all MPS types in patient-derived human cells instead of animal models—this might be particularly important when considering behavior that differs significantly between humans and any animal models), another point is the use of only one cell line of each MPS type. Since performing four biological repeats (necessary to obtain reliable results) in transcriptomic experiments with 12 cell lines (11 MPS types/subtypes and a control) is a technical and economical challenge, it was impossible to test cells from several patients suffering from the same disease. Thus, when interpreting the results, one must consider the existence of genetic variability between patients within every single MPS type. Therefore, it was crucial to find common changes in expression of genes in many MPS types relative to the control.

Such results support the interpretation that observed changes are disease-specific rather than resulting from random fluctuations. Finally, as in any experiment in which cell cultures are employed, one can observe differences in results between particular biological repeats. These differences arise from minor variations in culturing conditions (which become considerable when multiple) or the number of cell line passage. Therefore at least four biological repeats for each cell line were required to make reliable conclusions which had to be supported by detailed statistical analyses, performed according to commonly accepted procedures.

In our specific analyses, we have first focused on behavior-related genes, chosen on the basis of the QuickGO data base and included in the term “behavior” (GO:0007610), which transcripts were significantly changed in most (six or more) MPS types/subtypes. These would represent some common features of MPS. There were eight such genes, discussed below.

The *OXTR* gene codes for oxytocin receptor [20]. Abnormalities in oxytocin-mediated cellular signaling have been reported to be associated with social impairments, including autism spectrum disorder and inability to recognize faces [20]. Such behavioral problems are common in neuronopathic MPS types, particularly in MPS III. In fact, Sanfilippo disease (MPS III) is often misdiagnosed as autism spectrum disorder, especially at relatively early stages of the disease [21]. In our transcriptomic analyses, changes in expression of *OXTR* were pronounced in all MPS III subtypes. Perhaps surprisingly, levels of *OXTR* transcripts were also changed in non-neuronopathic MPS types, indicating that although oxytocin receptor functions can be associated with behavioral changes in MPS, they are perhaps not essential or causative but rather modulatory. On the other hand, it is tempting to speculate that more detailed observations of MPS patients in this regard might be substantiated.

The *OXTR* gene codes for oxytocin receptor [20]. Abnormalities in oxytocin-mediated cellular signaling have been reported to be associated with social impairments, including autism spectrum disorder and inability to recognize faces [20]. Such behavioral problems are common in neuronopathic MPS types, particularly in MPS III. In fact, Sanfilippo disease (MPS III) is often misdiagnosed as autism spectrum disorder, especially at relatively early stages of the disease [21]. In our transcriptomic analyses, changes in expression of *OXTR* were pronounced in all MPS III subtypes. Perhaps surprisingly, levels of *OXTR* transcripts were also changed in non-neuronopathic MPS types, indicating that although oxytocin receptor functions can be associated with behavioral changes in MPS, they are perhaps not essential or causative but rather modulatory. On the other hand, it is tempting to speculate that more detailed observations of MPS patients in this regard might be substantiated.

Expression of the *ITGA3* gene, coding for integrin alpha 3 subunit, is upregulated in seven types of MPS, both neuronopathic and non-neuronopathic. Mutations in the *ITAG3* gene were reported to be associated with interstitial lung disease, nephrotic syndrome, and epidermolysis bullosa [22,23]. No symptoms related to these disorders were reported as specific to MPS. It is likely that association of dysfunction of the integrin alpha 3 subunit to behavior is indirect, and connected to severe somatic symptoms. Moreover, in all MPS types, expression of *ITAG3* is enhanced, while above mentioned diseases are associated with mutations in this gene.

The *HRH1* gene encodes histamine receptor H1 [24]. Apart from association of this receptor in allergic rhinitis, causing tinnitus, snoring and rhinorrhea, recent studies indicated elevated expression of *HRH1* in patients with autism spectrum disorder [24]. Enhanced expression of this gene was also found by our transcriptomic analyses in several MPS types, including neuronopathic types I, II, III and VII. In fact, patients suffering from MPS often develop snoring and rhinorrhea, and symptoms mimicking autism spectrum disorder occur often, particularly in MPS III [21]. Therefore, up-regulation of the expression of the *HRH1* gene in cells derived from MPS patients also correlates well with this disorder, suggesting that elevated levels of histamine receptor H1 may contribute to behavioral changes observed in such patients.

The eIF4A3 (eukaryotic initiation factor 4A3), encoded by the *EIF4A3* gene, is a part of the exon junction complex [25]. Deficiency in this factor results in various neurodevelopmental disorders, including microcephaly [25]. Moreover, eIF4A3 has been implicated in the control of synaptic plasticity as well as development of learning and memory. It has been suggested that proper dosage of components of the exon junction complex is required for normal development and functions of neurons [26]. Therefore, one might assume that changes in expression of the *EIF4A3* gene, observed in our transcriptomic analyses, can contribute to mental retardation of MPS patients, particularly in learning and memory deficits. 

Expression of the *ID2* gene is significantly enhanced in six MPS types/subtypes, as revealed by our transcriptomic analysis. This gene codes for the inhibitor of DNA binding 2, which is a transcription factor capable of inhibition of oligodendrogenesis and oligodendrocyte precursor cell differentiation [27]. Hence, one might speculate that at least some neuronopathic symptoms occurring in MPS are related to up-regulation of the *ID2* gene expression. Ewing sarcoma, which is manifested with fever and visual disturbance, has been reported to be correlated with up-regulation of ID2 [28], and in fact, vision deficiency is common in MPS.

Homer 2 is a scaffolding protein which interacts with calcium-signaling proteins in cells [29]. Elevated levels of transcripts of the Homer 2-encoding gene, *HOMER2*, were detected in several MPS cell lines. *HOMER2* function has been associated with deafness [29], and hearing deficiency is common in MPS patients. Moreover, recent report suggested connection of Homer 2 is the regulation of basal anxiety [30], and anxiety-related behavioral problems occur frequently in MPS patients. Therefore, we suggest that changed expression of *HOMER2* may contribute to such MPS symptoms. Intriguingly, Homer-1 protein levels were found to be decreased in the brains of MPS I, MPS IIIA and MPS IIIB mice [31]. One might interpret these results as being in contrast to our transcriptomic analysis. However, there are several differences between both studies which can considerably influence such a comparison. First, we report the increased level of transcripts of the *HOMER2* gene, encoding Homer-2 protein, while previous studies [31] demonstrated decreased levels of the Homer-1 protein. Although belonging to the same family, Homer-1 and Homer-2 are actually two different polypeptides [32]. Second, human-derived and mouse cells were used for analyses performed by us and Wilkinson et al. [31], respectively, thus, inter-species differences can be considered. Third, we have determined mRNA levels while Wilkinson et al. [31] tested protein levels, thus, one might speculate that the differences could partially arise from specific translation regulation.

The *B2M* gene expression is down-regulated in MPS cells (Figure 3). This gene encodes β-2-microglobulin which is involved in surveillance and modulation of the immune response [33]. Dysregulation of β-2-microglobulin is correlated with various cancers, as well as recurrent infections [33]. Although the latter symptoms are very common in MPS, it appears that effects of impaired expression of *B2M* on behavior are indirect, arising from changed behavior of infected organism rather than direct disturbance of neuronal functions. 

Expression of the gene coding for insulin receptor (*INSR*) is down-regulated in MPS cells (Figure 3). The obvious consequence of this receptor deficit is diabetes [34], the disorder that often occurs also in MPS patients, particularly in MPS III, and in fact, impaired expression of *INSR* is evident in cells from patients suffering from all MPS III subtypes. However, impaired functions of IGF1 (insulin/insulin-like growth factor) signaling, which includes insulin receptor substrates, have been recognized as a risk factor for dementia and cognitive impairment [35]. Therefore, one might speculate that decreased levels of the insulin receptor may facilitate development of such symptoms in neuronopathic forms of MPS. 

To summarize the effects of changes in expression of genes which occur in many MPS types, it can be underlined that effects of such dysregulations may be associated with processes leading to well-known symptoms observed in MPS patients. Somewhat similar symptoms occur in diseases directly connected to mutations in corresponding genes (see Table 4 for summary). However, it seems unlikely that changes in gene expression are sole or predominant causes of behavioral disturbances in MPS, though one might assume that they can considerably modulate symptoms related to behavior.

We have also analyzed genes which expression is particularly significantly changed (log_2_ FC > 2.5) in specific MPS types/subtypes (Figure 4, Figure 5 and Figure 6). Below we discuss these genes and potential meaning of changes in levels in their transcripts for development of symptoms specific for particular MPS types. 

In MPS I, *MME* and *CAPN2* genes are significantly down-regulated, while *ID2* is significantly up-regulated (Figure 6). The *ID2* gene has been discussed above, among genes which expression is changed in many MPS types. The *MME* gene codes for membrane metalloendopeptidase, one of enzymes whose dysfunction is associated with Charcot-Marie-Tooth neuropathy [36]. Progressive weakness of muscles is characteristic for this disease, and occurs also in MPS I. The *CAPN2* gene encodes calpain 2, a calcium-dependent protease which function has been proposed to be associated with synaptic plasticity, and its dysregulation may lead to neurodegeneration [37]. Therefore, it is likely that significant down-regulation of *CAPN2* expression can contribute to neurodegeneration and behavioral symptoms in MPS I. 

In MPS II, apart from a significant increase in the *ID2* gene expression, the only gene characterized by significantly lowered levels of mRNA was *THBS1*. This gene codes for thrombospondin 1, an extracellular matrix protein which, due to interactions with various ligands, can regulate various processes [38]. One of effects of dysfunction of this protein is impaired vision, the symptom expressed also in MPS II.

MPS IIIA is the second type (after MPS IX) in terms of number of genes which revealed particularly significant changes in levels of expression. Apart from up-regulation of *OXTR*, discussed above together with other genes whose expression is changed many MPS types, following genes were down- or up-regulated significantly (log_2_ FC > 2.5) in MPS IIIA cells: *PTN, RCAN1, APOE,* and *MME*. The *MME* gene has been discussed in the paragraph devoted to MPS I. The *PTN* gene codes for pleiotrophin, a growth factor involved in the regulation of angiogenesis [39]. Although this process can be affected in MPS III, its effects on behavior can be indirect rather than primary. The regulator of calcineurin 1 is encoded by *RCAN1* [40], a gene down-regulated in MPS IIIA. This is in contrast to Down syndrome and Alzheimer’s disease, where overexpression of *RCAN1* has been reported [40]. On the other hand, it was demonstrated that short term stimulation of expression of this gene resulted in neuroprotection due to activation of expression of pro-survival genes [40]. Therefore, one may speculate that impairment of stress responses due to down-regulation of *RCAN1* may lead to dysfunctions of neurons and enhancement of behavioral disturbances observed in MPS IIIA. The *APOE* gene codes for apolipoprotein E, a multifunctional protein involved in lipoprotein metabolism [41]. There are multiple effects of changes in *APOE* expression and in functions of the gene product, including neuronopathy and neurodegeneration [41]. Therefore, a significantly decreased level of expression of *APOE* in MPS III can be associated with behavioral symptoms occurring in MPS IIIA. In fact, behavioral effects of changes in expression of genes coding for multifunctional proteins is usually very difficult to elucidate which is exemplified here not only by *APOE* but also by some other genes, like *SERPINE2* [42] (see below; discussion on MPS IX). 

In MPS IIIB, the *OXTR* gene is up-regulated as it is in many other MPS types. However, levels of *UCHL1* mRNA were also elevated very significantly. The *UCHL1* gene codes for ubiquitin *C*-terminal hydrolase L1 [43], an enzyme involved in deubiquitination, thus, playing a role in the control of protein degradation by proteasomes. Among all members of ubiquitin *C*-terminal hydrolases, the *UCHL1* gene product is especially abundant in the brain while occurring at low levels in somatic tissues [44]. It is required for the maintenance of axonal integrity, thus, playing a key role in functions of neurons. Mutations in the *UCHL1* gene have been associated with neurodegeneration [44]. Although we detected highly increased levels of *UCHL1* mRNA in MPS IIIB fibroblasts, another report demonstrated previously decreased levels of the gene product in several lysosomal storage diseases, including sialidosis, galactosialidosis, G_M1_-gangliosidosis, MPS IVA, MPS IVB, and Gaucher disease [45]. However, MPS III cells were not studied in that work, and we did not detect particularly highly changed levels of *UCHL1* mRNA in MPS IV (in fact, previous results demonstrated rather small, though significant, changes in abundance of ubiquitin *C*-terminal hydrolase L1 in MPS IVA and MPS IVB). Therefore, there is no inconsistency between results reported here and in the previously published article [45].

In MPS IIIC, apart from highly elevated levels of *ID2* and *OXTR* transcripts, occurring also in many other MPS types and described above, the *ALDH1A3* and *GAL* genes were down- and up-regulated, respectively. The *ALDH1A3* gene product is the aldehyde dehydrogenase 1A3 [46]. This enzyme is involved in production of retinoic acid, particularly through oxidizing all-trans-retinal [46,47]. Therefore, its deficiency results in impairment of eye formation and functions. In this light it is worth reminding that impaired vision occurs frequently in MPS patients. Galanin is the product of the *GAL* gene [48]. This neuropeptide has been demonstrated as a factor related to affective disorders and epilepsy [48]. Although dependence of MPS IIIC patients on alcohol, nicotine or opiate has never been described, due to severe neurodegeneration which precludes development of such disorders, epilepsy is common in this MPS type.

In MPS IIID, particularly significant changes in expression was found for *MME* (down-regulation), and *ID2* and *OXTR* (up-regulation) genes. These genes have been discussed in previous paragraphs.

Similarly, in MPS IVA, expression of previously discussed genes was particularly significantly changed. Namely, the *THBS1* gene was down-regulated and *ID2* was up-regulated, exactly as in MPS II. Note that no behavioral disorders were reported in this MPS type.

*OXTR* was up-regulated (as in many other MPS types) in MPS IVB. Moreover, up-regulation of *EPHB2* was also evident. This gene codes for the ephrin receptor B2. Its function is important for normal neuronal cell functions [49]. Since ephrin receptor B2-dependent signal transduction is involved in neuroprotection, changes in its amount might suggest occurrence of a response to specific cell dysfunctions.

MPS VI is characterized by up-regulation of *GAL* and *ID2.* Such changes in the expression regulation have been described above. In MPS VI, no behavior-related symptoms were reported.

Expression of three genes was particularly significantly up-regulated in MPS VII. All of them, *OXTR, ITGA* and *ID2* were discussed in previous paragraphs. 

In MPS IX, six genes revealed particularly significantly changed expression. Three of them were discussed above: *OXTR, GAL,* and *ID2* (all up-regulated). SERPINE2, encoded by the gene of the same name (*SERPINE2*, which is up-regulated in MPS IX) forms a complex with protease nexin-1, called SERPINE2/PN1 [42]. Multiple functions of this complex have been described, including enzyme inhibition, glycosaminoglycan binding, and modulation of cell surface receptors [42]. However, further studies are required to learn about specific functions of SERPINE2/PN1, and possible connections of overexpression of *SERPINE2* with symptoms occurring in MPS IX. The *VLDLR,* which is up-regulated in MPS IX, codes for a lipoprotein receptor family which bind apolipoprotein E [50]. Therefore, effects of dysregulation of *VLDLR* expression in MPS might be assumed to be associated to those for *APOE*. Interestingly, expression of *APOE* and *VLDLR* is regulated in opposite directions in MPS IX which can suggest a kind of compensatory cellular effects. *SLC7A11* codes for the cystine/glutamate antiporter solute carrier family 7 member 11 (known also as xCT). It is responsible for cystine uptake and glutathione biosynthesis [51]. Therefore, the *SLC7A11* gene product is involved in the protection of cells from oxidative stress. One might conclude that its elevated levels in MPS IX might indicate a cellular response to such a stress. Interestingly, although MPS IX is the disease type in which the highest number of behavior-related genes are up-regulated, no behavioral disorders were reported in patients. On the other hand, only very few patients suffering from MPS IX were described to date. Therefore, changes in the expression of such genes, reported here, suggest that these patients should be carefully observed in the light of their behavior. 

It should be indicated that transcriptomic studies were reported previously for MPS, but different models were used and only single MPS type was investigated in each individual study. When transcriptomes of MPS I patient fibroblast-derived iPSC neural stem cells were assessed, changes in expression of over 2500 genes in the severe MPS I subtype (MPS I-H), over 1600 genes in the intermediate subtype (MPS I-HS), and almost 700 genes in the “mild” subtype (MPS I-S), relative to control cells, were detected [52]. Although it is not easy to directly compare these results to those reported in this article, due to different cell types and cultivation conditions, it is worth noting that the above mentioned numbers are in the same range as the number of genes which expression is changed in MPS I in our analysis (733 genes). This corroborates the assumption that human fibroblasts might be a useful model in this type of studies. Another report demonstrated transcriptomic changes in the brain of MPS II mouse model [53]. Despite the different models and different species used in that study and this report, one should consider some common findings, reported in both articles, including significant changes in expression of genes clustered in certain GO terms, like locomotory behavior, learning or memory. This similarity may support the rationale of the use of human fibroblasts in preliminary transcriptomic analyses of behavior-related processes. In transcriptomic studies with brains of MPS VII mice [54], changes in expression of 853 genes, relative to control samples, were detected, which is comparable to the number indicated in this report (811 genes). Nevertheless, despite the very similar numbers reported in both studies, it is necessary to note that different significance levels were considered and number of genes with changed expression varied considerably between particular parts of the mouse brain [54]. On the other hand, it is tempting to propose that there are general similarities between the results of all these studies, suggesting that global changes in gene expression patterns may significantly contribute to development of MPS symptoms, including those related to behavior.

In summary, our transcriptomic studies indicated that expressions of various genes related to behavior are changed in MPS cells. Although we used patient-derived lines of fibroblasts, this model appears relevant for such studies, at least at the stage of preliminary identification of the phenomenon and indication of a set of genes which transcription can be down- and up-regulated in most or certain MPS types/subtypes. In fact, such a model has been used and accepted previously in studies on expression of behavior-related gene, *UCHL1,* in several lysosomal storage diseases [45]. In our study, we have distinguished genes whose expression is changed in most (six or more) MPS types, which indicate common features of this disease, as well as genes which are down- or up-regulated in specific MPS types, pointing to those which can contribute to specific behavioral changes in particular types/subtypes of this disease (the major results and relevant behavioral changes are summarized in Table 5). The major conclusion drawn from this work is that behavioral changes in MPS may arise not only from the primary storage of heparan sulfate, but also from secondary changes in expression of specific genes which are associated with various neurological processes. We suggest that although such changes in expression of genes cannot be solely responsible for changed behavior in MPS patients, they may contribute considerably in modulation of behavioral disturbances occurring in different MPS types. Because there are genes which expression is changed similarly in various MPS types, as well as genes with specific down- or up-regulated transcription in particular MPS types, it appears that there is no simple and direct correlation between the kind of stored GAG(s) and modulation of expression of genes related to behavior. Definitely, the processes leading to common and specific changes in expression of these genes in cells of patients suffering from various MPS types are more complex than a direct response to storage of particular GAG(s). Although it is obvious that further studies are required to identify molecular mechanisms of behavioral changes in MPS patients, this work suggest a possible way to find bases for understanding such mechanisms, especially as until now they remained almost completely unknown.

## 4. Materials and Methods

### 4.1. Cell Lines

In this work, cells of all MPS types/subtypes were tested, namely types I, II, IIIA, IIIB, IIIC, IIID, IVA, IVB, VI, VII, and IX. MPS I and MPS II fibroblasts derived from patients suffering from neuronopathic forms of these diseases. Control cell line (HDFa) was used in all transcriptomic analyses. Non-transformed, patient-derived lines of MPS fibroblasts and HDFa were purchased from the NIGMS Human Genetic Cell Repository at the Coriell Institute for Medical Research. In addition to HDFa, two other healthy fibroblast lines were employed in some RT-qPCR experiments (a line with no mutation detected, and an *IDUA* heterozygote); these lines were described previously [55,56] (all lines are characterized in Table 2). Fibroblasts used in this work had normal karyotypes. Cells were cultured in vitro in the DMEM medium (Dulbecco’s Modifies Eagle Medium) which was supplemented with antibiotics and 10% fetal bovine serum (FBS). When indicated, iduronate-2-sulfatase (Elaprase; Shire, Lexington, MA, USA) was added to final concentration 0.5 mg/L. Standard cultivation conditions were employed, i.e., 37 °C, 95% humidity, and 5% CO_2_ saturation.

### 4.2. RNA Isolation and Purification

Fibroblasts (5 × 10^5^ cells) were cultured on 10 cm-diameter plates. They were allowed to attach to plate walls overnight. Guanidine isothiocycanate and β-mercaptoethanol were used for cell lysis, employing the QIAshredder column. RNA extraction was performed using the RNeasy Mini kit (Qiagen, Hilden, Germany) and Turbo DNase (Life Technologies, Carlsbad, CA, USA), as described in instructions of manufacturers. RNA samples were tested for quality in the Agilent 2100 Bioanalyzer System with RNA Nano Chips (Agilent Technologies, Santa Clara, CA, USA).

### 4.3. RNA-seq Analysis

Illumina TruSeq Stranded mRNA Library Prep Kit was used for preparation of mRNA libraries. Following reverse transcription, libraries of cDNAs were sequenced using HiSeq4000 (Illumina, San Diego, CA, USA); following parameters were applied: 150 bp paired-end and minimum 4 × 10^7^ raw reads, giving a minimum of 12 Gb of raw data per each sample. FastQC v. 0.11.7 was used for quality assessment. RNA-seq data were deposited at NCBI Sequence Read Archive (SRA), with accession no. PRJNA562649. Hisat2 v. 2.1.0 program was used for mapping of raw readings to the GRCh38 human reference genome (from the Ensembl browser). Cuffquant and Cuffmerge softwares (v. 2.2.1) and the GTF Homo_sapiens.GRCh38.94.gtf file (from the Ensembl browser, https://www.ensembl.org/index.html as of 10 October 2019) were used to calculate the expression levels of transcripts. The Cuffmerge program was started with the library-norm-method classic-fpkm parameter normalizing the expression values by means of the FPKM (Fragments Per Kilobase Million) algorithm. Statistical significance was assessed using one-way analysis of variance (ANOVA) on log_2_(1 + x) values which have normal continuous distribution. The Benjamini–Hochberg method was used to estimate the false discovery rate (FDR). For comparisons between two groups, post hoc Student’s *t*-test with Bonferroni correction was employed. All statistical analyses were performed using R software v. 3.4.3. Differences were considered significant when *p* < 0.1 Transcript annotation and classification was performed using the BioMart interface for the Ensembl gene browser (https://www.ensembl.org/info/data/biomart/index.html, as for 10 October 2019).

### 4.4. RT-qPCR Analysis

In order to measure mRNA levels of selected genes, reverse transcription–quantitative real time PCR (RT-qPCR) was employed. Total RNA was subjected to reverse transcrption using iScript Reverse Transcription Supermix for RT-qPCR (BioRad, Hercules, CA, USA), according to the manufacturer’s instruction. RT-qPCR was carried out using primers descrived in Table 6. The mRNA levels were estimated using the 2^−ΔΔC(T)^ method. *GAPDH*, *G6PD* were used as reference genes (since no significant changes were detected between expressions of reference genes, results for tested genes are demonstrated relative to *GAPDH* transcript levels). One-way ANOVA was used to test statistical significance of differences between patient-derived fibroblasts and control cells. Differences were considered significant when *p* < 0.05.

## Figures and Tables

**Table 1:** Types of MPS with cognitive and behavioral symptoms indicated (based on references [2,4,5,6,7,8,9,10,11]).

MPS Type	Stored GAG(s)	Behavioral Symptoms
MPS I	Heparan sulfate, Dermatan sulfate	Cognitive decline (in some patients), fearful behavior, attention problems, low self-esteem, depression, social withdrawal
MPS II	Heparan sulfate, Dermatan sulfate	Cognitive decline (in most patients), hyperactivity, challenging behaviors, frustration, impulsivity, perseverative chewing behavior, seizure-like behavior, staring, myoclonus episodes, seizures, sleeping problems (reduced rapid eye movement sleep, night-time wakening, difficulty settling, insomnia), depression, social withdrawal
MPS III A,B,C,D	Heparan sulfate (less efficient secondary storage of dermatan sulfate)	Hyperactivity, expression of frustration, aggressive-like behavior, decreased attention, intellectual decline, autistic-like behaviors, primarily social and emotional abnormalities, reduced fear, sleep disturbances (daytime sleepiness, difficulties settling, night-time and early morning wakening, insomnia, laughing and singing during the night), disturbances in the circadian rhythm
MPS IV A,B	Keratan sulfate, Chondroitin sulfate	None
MPS VI	Dermatan sulfate	None
MPS VII	Heparan sulfate, Dermatan sulfate, Chondroitin sulfate	Mental retardation
MPS IX	Hyaluronic acid	None ^a^

^a^ Only a very few patients were described in the literature to date, thus, no solid conclusions can be drawn in this matter [2]. MPS—mucopolysaccharidosis; GAG—glycosaminoglycan

**Table 2:** Characteristics of fibroblast lines derived from MPS patients and control cell lines.

Cell Line	Race *	Sex *	Age *^#^	Mutated Gene and Its Locus *	Mutation *	Catalog Number *
MPS I	Caucasian	Female	1	*IDUA*, 4p16.3	Homozygote p.Trp402Ter/p.Trp402Ter	GM00798
MPS II	Caucasian (ethnicity: Haitian)	Male	3	*IDS*, Xp28	Hemizygote p.His70ProfsTer29	GM13203
MPS IIIA	Caucasian	Female	3	*SGSH*, 17q25.3	Complex heterozygote p.Glu447Lys/p.Arg245His	GM00879
MPS IIIB	Caucasian	Male	7	*NAGLU*, 17q21	Homozygote p.Arg626Ter/p.Arg626Ter	GM00156
MPS IIIC	Unknown	Male	8	*HGSNAT*, 8p11.1	Not determined	GM05157
MPS IIID	Asian Indian	Male	7	*GNS*, 12q14	Homozygote p.Arg355Ter/p.Arg355Ter	GM05093
MPS IVA	Caucasian (ethnicity: Mexican)	Female	7	*GALNS*, 16q24.3	Not determined	GM00593
MPS IVB	Caucasian	Female	4	*GLB1*, 3p22.3	Complex heterozygote p.Trp273Leu/p.Trp509Cys	GM03251
MPS VI	Black	Female	3	*ARSB*, 4q14.1	Not determined	GM03722
MPS VII	African American	Male	3	*GUSB*, 7q21.11	Complex heterozygote p.Trp627Cys/p.Arg356Ter	GM00121
MPS IX	Unknown	Female	14	*HYAL1*, 3p.21.3	Not determined	GM17494
Control line 1 (HDFa)	Unknown	Male	Adult	N/A	N/A	N/A
Control line 2	Caucasian	Female	41	N/A	N/A	N/A
Control line 3 (*IDUA* heterozygote)	Caucasian	Male	51	*IDUA*, 4p16.3	Heterozygote WT/p. Δ349	N/A

* according to cell line description in Coriell Institute; ^#^ age (years) at the time of cell collection; N/A—not applicable.

**Table 3:** Number of up- and down-regulated transcripts of genes related to behavior (term GO:0007610 in the QuickGO database) in different types of MPS relative to control cells (HDFa).

Transcripts	Number of Significantly Changed Transcripts in Particular MPS Type vs. HDFa Line										
	I	II	III A	III B	III C	III D	IVA	IVB	VI	VII	IX
Up-regulated	12	7	16	13	15	13	4	16	5	17	21
Down-regulated	9	4	23	13	7	8	6	13	3	8	8
Total	21	11	39	26	22	21	10	29	8	25	29

**Table 4:** Changes in expression (transcripts’ levels) of particular genes in MPS types are depicted, indicating log_2_ fold change (FC) relative to control cells (HDFa); up-regulation is marked in bold and down-regulation in marked in italic, while not statistically significant differences are not marked.

Transcript	log_2_FC of Selected Transcripts in Particular MPS Type vs HDFa Line										
	I	II	III A	III B	III C	III D	IVA	IVB	VI	VII	IX
*OXTR*	4.08	1.67	**3.77**	**5.20**	**6.43**	**4.20**	5.61	**6.07**	3.13	**2.55**	**4.23**
*ITGA3*	**1.63**	0.40	**1.46**	**2.46**	**1.98**	1.84	**2.15**	**1.56**	1.14	**2.63**	1.59
*HRH1*	**1.16**	**1.06**	0.63	0.41	**0.84**	**1.01**	0.57	**1.10**	0.76	**1.11**	**1.19**
*EIF4A3*	**1.21**	**1.05**	0.94	−0.68	**1.14**	0.22	**1.28**	**1.60**	0.96	−0.01	**0.88**
*ID2*	3.08	**3.32**	2.85	2.58	2.86	**3.45**	**3.00**	2.90	**3.11**	**3.32**	**3.91**
*HOMER2*	**1.48**	0.55	**1.66**	**1.82**	**1.81**	1.00	1.59	1.63	1.07	**2.27**	**1.58**
*B2M*	*−1.23*	−0.61	*−1.57*	*−1.82*	*−1.27*	−0.39	−1.05	*−1.37*	−0.25	−0.43	*−1.23*
*INSR*	−0.38	−0.54	*−0.41*	*−0.70*	*−0.66*	*−0.57*	*−0.42*	−0.34	−0.32	*−0.50*	−0.01

**Table 5:** Summary of transcriptomic studies of genes related to behavior (term GO:0007610 in the QuickGO database) changed significantly in cell lines derived from patients suffering from all types/subtypes of MPS.

Gene with Expression Changed in Various MPS Types	Gene Product	Disease(s) Associated with the Gene	Symptoms	Similar Symptoms in MPS
**Six or More MPS Types**				
*OXTR*	Oxytocin receptor	Autism spectrum disorder, prosopagnosia, adenomyosis	Autism, inability to recognize faces (prosopagnosia)	Autism, cognitive dysfunctions
*ITGA3*	Integrin subunit alpha 3	*Ilneb*	Interstitial lung disease, nephrotic syndrome, and epidermolysis bullosa	None reported
*HRH1*	Histamine receptor H1	Allergic rhinitis, autism spectrum disorder	Tinnitus, snoring and rhinorrhea, autism	Snoring and rhinorrhea, autism
*EIF4A3*	Eukaryotic translation initiation factor 4A3	Mandibulofacial dysostosis, Guion-Almeida type	Growth and mental retardation (learning and memory deficits), mandibulofacial dysostosis, microcephaly	Growth and mental retardation (learning and memory deficits), dysostosis multiplex
*ID2*	Inhibitor of DNA binding 2	Ewing sarcoma	Fever, visual disturbance and eye manifestations	Visual disturbance and eye manifestations
*HOMER2*	Homer scaffold protein 2	Deafness, autosomal dominant 68	Sensorineural hearing loss, anxiety	Hearing deficiency, anxiety
*B2M*	β-2-microglobulin	Immunodeficiency 43	Recurrent respiratory tract infections and severe skin disease	Recurrent respiratory tract infections and severe skin disease
*INSR*	Insulin receptor	Donohue syndrome, dementia and cognitive impairment	Insulin resistance, dementia and cognitive impairment	Diabetes, cognitive impairment
**MPS I (log_2_FC > 2.5)**				
*MME*	Membrane metalloendopeptidase	Axonal Charcot-Marie-Tooth Disease type 2t	Progressive weakness and atrophy of muscles	Progressive weakness of muscles
*CAPN2*	Calpain 2	Alzheimer Disease	Gradual loss of memory, judgment, and the ability to function socially	Gradual loss of memory and the ability to function socially
**MPS II (log_2_FC > 2.5)**				
*THBS1*	Thrombospondin 1	Thrombotic thrombocytopenic purpura	Bleeding problems, impaired vision	Impaired vision
**MPS IIIA (log_2_FC > 2.5)**				
*PTN*	Pleiotrophin	Macrodactyly	Fibrofatty tissue enlargement and bony overgrowth	None reported
*RCAN1*	Regulator of calcineurin 1	Down syndrome, Alzheimer’s disease	Dysmorphology, Mental retardation, hearing and vision problems	Mental retardation, hearing and vision problems
*APOE*	Apolipoprotein E	Lipoprotein glomerulopathy, hyperlipoproteinemia type III	Proteinuria, progressive kidney failure, tremor, angina pectoris and edema, neurodegeneration	Neurodegeneration
*MME*	Membrane metalloendopeptidase	Axonal Charcot-Marie-Tooth disease type 2t	progressive weakness and atrophy of muscles	progressive weakness of muscles
**MPS IIIB (log_2_FC > 2.5)**				
UCHL1	Ubiquitin *C*-Terminal Hydrolase L1	Spastic Paraplegia 79	gradual, progressive weakness and spasticity of the lower limbs, neurodegeneration	Weakness of muscles, neurodegeneration
**MPS IIIC (log_2_FC > 2.5)**				
*ALDH1A3*	Aldehyde dehydrogenase 1 family member A3	Microphthalmia type 8	Small size of a single eye to complete bilateral absence of ocular tissues	Impaired vision
*GAL*	Galanin and GMAP prepropeptide	Epilepsy, familial temporal lobe, type 8	Temporal lobe epilepsy	Epilepsy
**MPS IIID (log_2_FC > 2.5)**				
*MME*	Membrane metalloendopeptidase	Axonal Charcot-Marie-Tooth disease type 2t	progressive weakness and atrophy of muscles	progressive weakness of muscles
**MPS IVA (log_2_FC > 2.5)**				
*THBS1*	Thrombospondin 1	Thrombotic thrombocytopenic purpura	Bleeding problems	None reported
**MPS IVB (log_2_FC > 2.5)**				
*EPHB2*	Ephrin receptor B2	Prostate cancer/brain cancer susceptibility	Prostate and brain cancers	None reported
**MPS VI (log_2_FC > 2.5)**				
*GAL*	Galanin and GMAP prepropeptide	Epilepsy, familial temporal lobe, type 8	Temporal lobe epilepsy	None reported
**MPS VII (log_2_FC > 2.5)**				
None				
**MPS IX (log_2_FC > 2.5)**				
*SERPINE2*	Serpin family E member 2	Visceral heterotaxy	Abnormal distribution of the major visceral organs	None reported
*GAL*	Galanin and GMAP prepropeptide	Epilepsy, familial temporal lobe, type 8	Temporal lobe epilepsy	None reported
*VLDLR*	Very low-density lipoprotein receptor	Cerebellar ataxia, mental retardation, and dysequilibrium syndrome 1	Ataxia associated with an intellectual disability	None reported
*SLC7A11*	Solute carrier family 7 member 11	Dyscalculia	learning disability involving a math disability	None reported

**Table 6:** Primers used in RT-qPCR.

Gene	Forward Primer Sequence	Reverse Primer Sequence
*OXTR*	5′-CTGCTACGGCCTTATCAGCTT-3′	5′-CGCTCCACATCTGCACGAA-3′
*MME*	5′-AGAAGAAACAGCGATGGACTCC-3′	5′-CATAGAGTGCGATCATTGTCACA-3′
*THBS1*	5′-AGACTCCGCATCGCAAAGG-3′	5′-TCACCACGTTGTTGTCAAGGG-3′
*APOE*	5′-GTTGCTGGTCACATTCCTGG-3′	5′-GCAGGTAATCCCAAAAGCGAC-3′
*ALDH1A3*	5′-TGAATGGCACGAATCCAAGAG-3′	5′-CACGTCGGGCTTATCTCCT-3′
*SGK1*	5′-AGGATGGGTCTGAACGACTTT-3′	5′-GCCCTTTCCGATCACTTTCAAG-3′
*PENK*	5′-CGGTTCCTGACACTTTGCACT-3′	5′-CACATTCCATTACGCAAGCCA-3′
*UCHL1*	5′-CCTGTGGCACAATCGGACTTA-3′	5′-CATCTACCCGACATTGGCCTT-3′
*GAL*	5′-CCGGCCAAGGAAAAACGAG-3′	5′-GAGGCCATTCTTGTCGCTGA-3′
*GAPDH*	5′-GGAGCGAGATCCCTCCAAAAT-3′	5′-GGCTGTTGTCATACTTCTCATGG-3′
*G6PD*	5′-CGAGGCCGTCACCAAGAAC-3′	5′-GTAGTGGTCGATGCGGTAGA-3′

## Abbreviations

DMEM	Dulbecco’s Modifies Eagle Medium
FC	Fold change
FDR	False discovery rate
FPKM	Fragments Per Kilobase Million
GAG	Glycosaminoglycan
HS	Heparan sulfate
LSD	Lysosomal storage diseases
MPS	Mucopolysaccharidosis
RT-qPCR	Reverse transcription quantitative polymerase chain reaction
